# Antimicrobial Effect of Visible Light—Photoinactivation of *Legionella rubrilucens* by Irradiation at 450, 470, and 620 nm

**DOI:** 10.3390/antibiotics8040187

**Published:** 2019-10-15

**Authors:** Julian Schmid, Katharina Hoenes, Petra Vatter, Martin Hessling

**Affiliations:** Department of Medical Engineering and Mechatronics, Ulm University of Applied Sciences, Albert-Einstein-Allee 55, D 89081 Ulm, Germany; Julian.Schmid@thu.de (J.S.); Katharina.Hoenes@thu.de (K.H.); Petra.Vatter@thu.de (P.V.)

**Keywords:** legionella, *Legionella rubrilucens*, photoinactivation, disinfection, infection prevention, visible light, porphyrins, flavins

## Abstract

Despite the high number of legionella infections, there are currently no convincing preventive measures. Photoinactivation with visible light is a promising new approach and the photoinactivation sensitivity properties of planktonic *Legionella rubrilucens* to 450, 470, and 620 nm irradiation were thus investigated and compared to existing 405 nm inactivation data for obtaining information on responsible endogenous photosensitizers. Legionella were streaked on agar plates and irradiated with different doses by light emitting diodes (LEDs) of different visible wavelengths. When irradiating bacterial samples with blue light of 450 nm, a 5-log reduction could be achieved by applying a dose of 300 J cm^−2^, whereas at 470 nm, a comparable reduction required about 500 J cm^−2^. For red irradiation at 620 nm, no inactivation could be observed, even at 500 J cm^−2^. The declining photoinactivation sensitivity with an increasing wavelength is consistent with the assumption of porphyrins and flavins being among the relevant photosensitizers. These results were obtained for *L. rubrilucens*, but there is reason to believe that its inactivation behavior is similar to that of pathogenic legionella species. Therefore, this photoinactivation might lead to new future concepts for legionella reduction and prevention in technical applications or even on or inside the human body.

## 1. Introduction

*Legionella* species are widely distributed in natural and artificial water systems, such as lakes, rivers, building water distribution networks, and cooling towers, and any system producing aerosolized water represents a potential origin of legionellosis [1,2].

By inhaling aerosols of water contaminated with legionella, they can cause Pontiac fever or even lethal pneumonia. The lethality rate for *Legionella pneumonia* infections in Europe is about 8% [3] and based on a worldwide statistic, legionella are even among the ten most important lethal pathogens [4]. Therefore, legionella are widespread environmental infectious agents representing a hazard to public health. Suspected sources of legionella infections relevant to humans are, e.g., showers or cooling water reservoirs, but also dental instruments or flushing toilets that produce water aerosols [5,6].

For the prevention of legionella infections, many conventional disinfection procedures are possible: chlorine and ozone treatment or thermal disinfection. These measures are similar in that they all entail various disadvantages, such as the generation of toxic byproducts or high-energy consumption. In addition to thermal disinfection, UV-C irradiation is another broadly used non-chemical legionella disinfection method that is based on DNA and RNA damage due to the dimer formation of nucleotides, which interferes with protein biosynthesis [7]. However, UV-C radiation also poses a potential danger, if there is a risk of the irradiation of human tissue, because, in human cells, it can lead to mutagenic effects. In addition, water disinfection with UV-C radiation, which is usually generated in gas discharge lamps with a toxic mercury content, is complicated by strong UV-C absorption. Even in clear water, or other media of a high transmittance, the penetration depth of UV-C will only be in the range of 10 cm, and thus not suitable for the disinfection of deep water reservoirs. Visible light offers a much higher penetration depth in water and is relatively harmless to human tissue [8,9,10,11]. Its inactivation potency is about 1000 times weaker than that of UV-C radiation and is thus not suitable for applications with high flow rates, since the residence times are too short to reach adequate doses.

**Figure 1:**
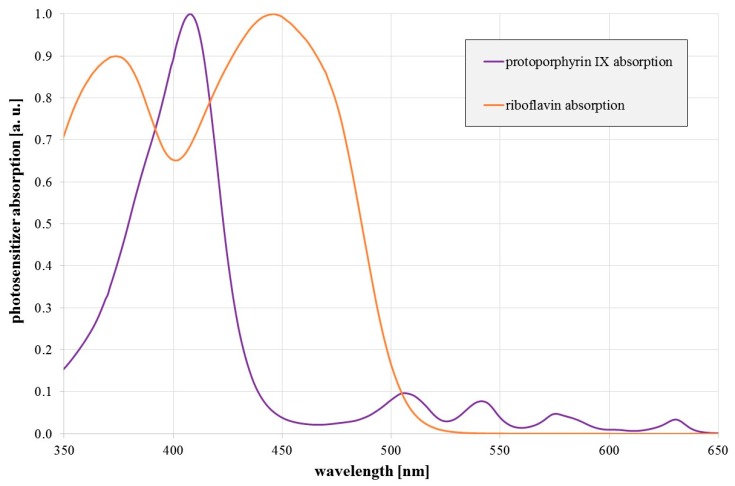
Relative absorption of protoporphyrin IX (in DMSO) and riboflavin (in H_2_O) as a function of wavelength.

The antimicrobial effect of visible light has been examined and confirmed by many different research groups within the last 15 years [12,13,14,15,16,17]. The effect is especially strong in the violet and blue spectral range. It is based on endogenous photosensitizers that absorb visible light and generate reactive oxygen species (ROS). These radicals attack various intracellular biomolecules, which may result in lethal damage. So far, the occurrence and involvement of potential endogenous photosensitizers is only partially understood. While porphyrins such as coproporphyrin III, protoporphyrin IX, or uroporphyrin III are believed to be the most important photosensitizers for violet radiation (400–420 nm) [12,13,15,18,19], flavins, like riboflavin, are regarded as possible photosensitizers responsible for the antimicrobial properties of blue radiation in the range of 450–470 nm [20,21,22]. Figure 1 illustrates a typical porphyrin and flavin absorption spectrum.

We have already demonstrated that legionella are sensitive to violet light of a wavelength of 405 nm [23], which is probably mainly caused by porphyrins. For other microorganisms, the application of blue light in the range of 450–470 nm has previously been proven successful in bacterial photoinactivation, but so far, it has never been tried on legionella. It is supposed that the sensitivity of bacteria in the blue wavelength range around 450 nm arises from the involvement of flavins as photosensitizers [22,24,25], but, until now, there has been little experimental evidence [26]. Therefore, all species have to be investigated separately in terms of their blue wavelength sensitivity. Since sensitivities to specific wavelengths vary for each species it is relevant to know the dose required to achieve sufficient reduction results for planning procedures for specific environments and their microbial species. According to various authors, whose results were collected in [14], *Porphyromonas gingivalis* needs an average dose of 47.5 J cm^−2^ between 450 and 455 nm, *Pseudomonas aeruginosa* requires 142.7 J cm^−2^, *Escherichia coli* requires 269 J cm^−2^, and *Staphylococcus aureus* requires 375.2 J cm^−2^ to reduce the colony-forming units by 1 log.

An investigation of the sensitivity of legionella towards this wavelength might lead to new applications for which UV-C radiation or even violet light is still too aggressive or lacks the desired penetration depth, which is longer in the blue spectral region. This is also true for human tissue and might offer therapeutic applications because, although visible light seems to be harmful to many bacteria, human cells appear to be more resistant [8].

It would be even more interesting if red light, e.g., around 620–630 nm, exhibits antimicrobial properties against legionella, because the penetration depth in human tissue is high at this wavelength and this could offer the possibility to treat Legionnaire’s disease with red light, even from the outside. Porphyrins show absorption here besides their main peak around 400 nm (Figure 1), which makes it a reasonable approach to investigate the impact of these red wavelengths.

Furthermore, a comparison of the photoinactivation sensitivities towards different irradiation wavelengths could deliver insights into legionella’s endogenous photosensitizers’ composition [26]. Due to safety and regulatory issues, the experiments presented here are based on the non-pathogenic legionella strain *Legionella rubrilucens*. For *L. rubrilucens*, no pathogenicity has been reported so far, although the occurrence of a co-infection was published in the past [27].

## 2. Results

### 2.1. Irradiation Experiments

**Figure 2:**
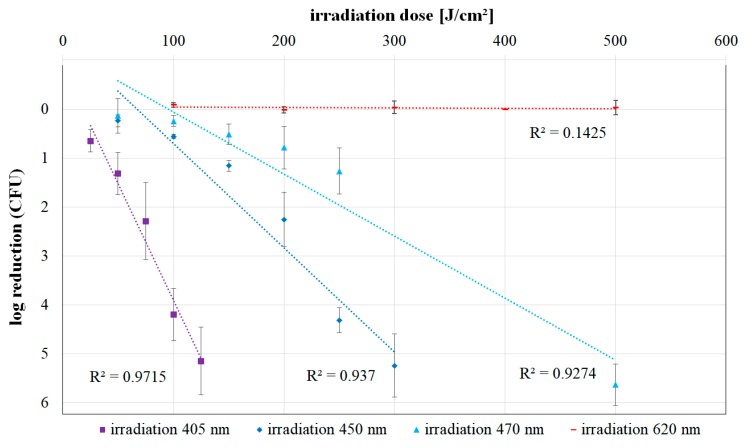
Log reduction of *Legionella rubrilucens* (DSM No. 11884) for increasing doses of 405 [23], 450, 470, and 620 nm irradiation. The values are given with the standard deviation for three independent individual runs per irradiation dose. In addition, a trend line of the values and the coefficient of determination *R*^2^ were created for each wavelength.

For the irradiation results at the wavelengths of 450, 470, and 620 nm, the applied irradiation doses and the corresponding observed mean colony-forming unit (CFU) reductions are presented in Table 1. The mean CFU reduction is composed of the reduction values of the individual trials for the respective dose. Figure 2 depicts this data together with an exponential trend line for each wavelength. The previously published 405 nm inactivation values of *L. rubrilucens* [23] are included in Figure 2 for comparison.

Exponential characteristics would be represented by straight lines in Figure 2, but for all applied wavelengths, except 620 nm, the graphs start with a bent curve, the so called “shoulder”—a region of lower sensitivity/reduced photoinactivation/reduced negative slope. For higher doses, the curves in Figure 2 become steeper and more linear, approaching the assumed exponential behavior.

It is apparent that the inactivation efficacy of the irradiation increases for shorter wavelengths. While no photoinactivation could be detected even after an irradiation with 500 J cm^−2^ at 620 nm, bacterial counts decreased by more than 5-log steps for the same irradiation dose at 470 nm. When irradiating with 450 nm, a 5-log reduction was achieved after approximately 300 J cm^−2^, and for 405 nm irradiation, this was reached with a dose of just 125 J cm^−2^.

Comparing the trend lines of the inactivation curves at 405, 450, and 470 nm for higher doses, the differences in efficiency are discernible: whereas at 405 nm an average of about 25 J cm^−2^ is required for one-log reduction, at 450 nm, it is about 60 J cm^−2^ and at 470 nm, it is about 100 J cm^−2^. This means that the violet radiation at 405 nm is about four times more efficient than the blue radiation at 470 nm. The impact of the 450 nm irradiation is between these wavelengths, with an efficiency difference of about 1.6 between 450 and 470 nm.

### 2.2. Colony Quantification

At the beginning of the incubation, no colonies can be detected, as is the case on the agar plates for all wavelengths. On the third day, the first colonies appear on the agar plates of the reference and 620 nm irradiation. On day five, clearly visible colonies are present for reference and 620 nm irradiation plates, while at shorter wavelengths, the colonies are significantly smaller. Even between 470, 450, and 405 nm, differences in colony sizes are observable on day five. For shorter wavelengths, the colonies become smaller until they are almost imperceptible at 405 nm. It can also be observed that in some cases, radiation-exposed samples develop colonies, which do not reach the size of the colonies of untreated samples, even after longer incubation periods.

Independent of their size, the number of colonies for each dose and wavelength is converted to the achieved log reduction. It should be mentioned that an extended incubation phase of 30 days did not result in any differences in colony numbers compared to the incubation of only 5 days.

### 2.3. Spectroscopic Investigations

**Figure 3:**
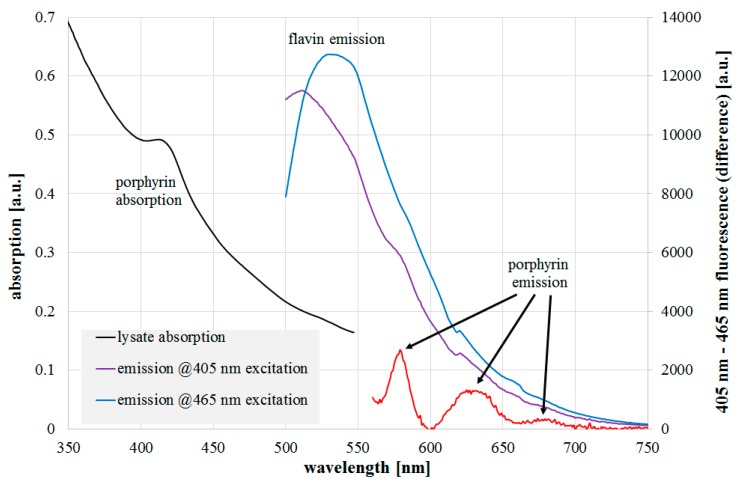
*L. rubrilucens* lysate absorption (black line), fluorescence emission at 405 nm (violet line), 465 nm excitation (blue line), and difference of normalized emissions (red line). The assumed peak origins are also given.

The results of absorption and fluorescence emission measurements of the *L. rubrilucens* lysate are presented in Figure 3. Within the absorption data, there is a peak at 410–420 nm with a spectral width of about 30 nm, which is in good agreement with typical porphyrin characteristics. Distinct flavin absorption is not recognizable, but that is not unexpected because of its broad absorption width of more than 100 nm. When the cell lysate is irradiated by a 465 nm laser near the flavin absorption maximum, a typical flavin fluorescence emission with a maximum of around 530 nm is observed. The 405 nm laser should also excite flavin fluorescence, but its wavelength is also capable of porphyrin excitation, so the resulting emission consists of simultaneous emissions of different fluorophores. To reduce the effect of the strong flavin emission, both emission spectra could either be divided or subtracted from each other to get more insight into the fluorophores besides flavins. In Figure 3, the spectra were normalized to each other and the resulting fluorescence differences revealed emission peaks at around 580, 630, and 675 nm in the emission for the 405 nm excitation, which were probably caused by one or more of the following porphyrins: protoporhyrin IX, coproporphyrin III, uroporphyrin III, or zinc protoporphyrin IX. Their absorption and emission spectra are all well-known from the literature and our own measurements. As mentioned above, the first three porphyrins are assumed to be the most relevant for bacterial photoinactivation. The latter was observed as a fluorescence peak in [21,28,29]; however, it was not identified by the authors and was only recently put into context with the photoinactivation potency [26]. Comparing the detected peaks in the legionella lysate, the peak at 630 nm finds a good match with protoporphyrin IX (measured main fluorescent peak at 631 nm). The peak at 580 nm cannot be assigned to the most often named candidates, but to the metalloporphyrin zinc protoporphyrin.

## 3. Discussion and Conclusions

The observed deviation of the exponential bacterial reduction for lower irradiation doses in Figure 3, the so-called “shoulder”, and its wavelength dependence, are in good agreement with the photoinactivation literature results presented for other microorganisms. For example, Webb et al., described a similar increase of the shoulder effect with an increasing wavelength for experiments with *Escherichia coli* [30]. They also formulated the hypothesis that the shoulder effect itself could be explained by dose-dependent repair processes and that the repair processes would become insufficient with increasing doses [30].

For the irradiation with red wavelengths around 620 nm, with its low disinfection success, Sailer et al., obtained similar results [31]. They irradiated a *Pseudomonas* suspension with a 630 nm laser and doses of up to 400 J cm^−2^. In addition, the group raised the porphyrin content of the *Pseudomonas* by adding 5-aminolevulinic acid to the culture medium. Despite this procedure, they did not observe any inactivation effect, neither for incubated *Pseudomonas* in the growth phase, nor for unincubated controls. Only the irradiation of *Pseudomonas* in the stationary phase achieved a reduction of the CFU number of 25% [31].

A possible explanation for the missing antimicrobial effect at 620 nm radiation could be the absorption ratio of the porphyrins between 405 and 620 nm, which differ by more than one order of magnitude. Much higher doses are probably required to achieve a detectable antimicrobial effect at 620 nm.

The high coefficients of determination at 405, 450, and 470 nm show a clear correlation between the logarithmic decrease of CFU and the applied irradiation dose. Therefore, the efficiency differences between the wavelengths can be well-represented by exponential trend lines, at least for higher doses.

The differences in efficiency between 405 and 470 nm irradiation are consistent with the observations of Hessling et al. In their study, the necessary reduction values were about two to five times higher for 470 nm compared to 405 nm irradiation [14].

Up to 20% of the higher photoinactivation efficiency of 450 nm compared to 470 nm radiation can be explained by the lower flavin absorption at 470 nm. The remaining 20% difference could originate in a residual porphyrin influence because the above mentioned porphyrins (protoporphyrin IX, coproporphyrin, uroporphyrin, or zinc protoporphyrin) do not have strong absorptions at 450 nm, but their absorption at 470 nm is even considerably lower by about a factor of two. For a quantitative analysis of the differences between the impacts of different irradiation wavelengths, more spectral data would be necessary. This would allow a new analysis approach to be performed that is able to deliver photoinactivation contributions of different photosensitizers for each irradiation wavelength [26].

A photographic documentation of the agar plates after irradiation—not depicted here—showed delayed growth for all the blue/violet wavelengths compared to the non-irradiated control. There seems to be a correlation between the efficiency of a wavelength and the incubation time required until colonies become detectable. A trigger for such an occurrence could be the amount of reactive oxygen species or oxidative stress, which initially prevents *L. rubrilucens* from replicating due to the intracellular damage of biomolecules [32]. After a certain adaptation or regeneration phase, these legionella resume replication and form colonies. The extent of colony formation delay during incubation may be an indicator of the amount of induced oxidative stress.

The results of the presented irradiation experiments are based on *L. rubrilucens* and not on the more important pathogenic *L. pneumophila*. However, for other bacterial species, it was observed that related strains exhibit similar doses for a log reduction, like *Listeria ivanovii* at 44.9 J cm^−2^, *Listeria monocytogenes* at 45.9 J cm^−2^, and *Listeria seeligeri* at 55.9 J cm^−2^, or *Mycobacterium smegmatis* at 67.8 J cm^−2^ and *Mycobacterium terrae* at 57.6 J cm^−2^ [14]. Additionally, Fila et al., investigated the photoinactivation sensitivity for about 20 *P. aeruginosa* strains and observed similar sensitivities for different strains, independent of their virulence/antibiotic resistance properties [33]. Therefore, we expect *L. pneumophila* to have similar photoinactivation properties to *L. rubrilucens*. It should also be mentioned that this investigation was performed with planktonic bacteria. Legionella within host cells might exhibit different photoinactivation properties.

It is conceivable that the observed legionella reductions were not only caused by intracellular ROS, but also by radicals or toxins from the outside generated by agar irradiation, because the charcoal yeast extract agar contains riboflavin, which is capable of the generation of ROS, like hydrogen peroxide, during irradiation. This would be in accordance with the literature results [34,35,36,37] that describe observed changes in the growth behavior of microorganisms for light irradiated media. All authors concluded that the main influence was caused by riboflavin and its H_2_O_2_ generation. Unfortunately, they used different culture media, investigated different bacteria, and did not give much information on the spectrum or dose of their irradiation. Therefore, it is difficult to draw direct conclusions from these papers for our legionella experiments on charcoal yeast agar and for our wavelengths and irradiation doses. Trzaska et al., however, performed a similar experiment with more information on the irradiation [38]. They inactivated fungal pathogens on different types of agar with blue/violet light. To detect a potential agar-caused artefact, they irradiated some agar plates prior to the photoinactivation experiments. They applied roughly similar excitation wavelengths, doses, and agar as have been presented in this paper: 216 J cm^−2^ at 405 nm on yeast extract agar (without charcoal). They observed no difference between non-irradiated and irradiated agar plates. This indicates a rather low influence of the violet/blue irradiated yeast extract agar.

Even more important is the fact that the yeast extract agar that was employed in this study additionally contained charcoal. Charcoal does not only exhibit its well-known adsorption/detoxification characteristics, but also has a strong H_2_O_2_ decomposition capability, which was even investigated for different legionella strains on charcoal yeast extract medium [39]. Therefore, it can be assumed that even if ROS are generated by the agar irradiation, their concentration is effectively reduced by the charcoal yeast extract agar and the influence of the irradiated agar on the legionella survival is low.

Based on the data for 620 nm, the idea of treating Legionnaire’s disease with red light seems to be unrealistic. In contrast, 450 and 470 nm radiation exhibit a clear antimicrobial effect. There are possible permanent applications for continuous use based on particularly powerful, cost-effective, and energy-efficient LEDs. Applications for blue irradiation could be various types of water depots, such as water dispensers, water fountains, or even large water tanks in cooling systems [23]. Blue radiation seems to be advantageous for contamination prevention as opposed to conventional disinfection measures with chlorine or ozone. There are no negative effects like toxic by-products or continuously incurring chemical consumables and associated consumable costs. In addition, due to the efficiency in the application of blue radiation, the energy consumption is low, in contrast to the high-energy requirement that is incurred during thermal disinfection, for example. Even therapeutic applications of visible light on or in humans seem to be possible, since the sensitivity of human cells is much lower than that of bacteria such as legionella [9]. For example, one possible future application could be the insertion of a blue light source into the lung to treat or prevent lung infections such as those caused by *Legionella pneumonia* [23].

## 4. Materials and Methods

### 4.1. Bacterial Preparation

*Legionella rubrilucens* was obtained from ‘The Leibniz Institute DSMZ—German Collection of Microorganisms and Cell Cultures GmbH’ (Braunschweig, Germany) as freeze-dried samples (DSM No. 11884). From the primary stock, a plate of smeared bacteria was prepared on glycine vancomycin polymyxin B cycloheximide agar (GPVC) [40]. With a colony of this plate, 3 mL of liquid buffered yeast extract (BYE) medium was inoculated as a preculture, heat treated for 30 min at 50 °C to reduce possible contaminations, and incubated at 37 °C on an orbital shaker (200 rpm) for 20 h. The bacteria were harvested by centrifugation at 4800× *g* for 5 min. After discarding the supernatant, the pellet was resuspended in phosphate buffered saline (PBS, 0.0119 M). For further propagation, the desired amount of bacterial suspension was inoculated in the appropriate volume (5 mL) of fresh buffered yeast extract medium (BYE) [41] until an optical density of 0.1 at a wavelength of 600 nm was achieved, and was subsequently incubated at 37 °C for 4 h. After the main culture had reached an OD600 of approximately 0.3, three samples of 1000 µL each were taken and washed in PBS by pelletizing them at 4800× *g* for 5 min in a centrifuge and resuspending them in 1000 µL PBS. Three decimal dilution series were respectively prepared, with an initial concentration of approximately 10^8^ CFU mL^−1^. For irradiation experiments, legionella were evenly plated on petri dishes with buffered charcoal yeast extract (BCYE) agar, whose surface was dried for 1–1.5 h prior to the irradiation. The choice of a suitable dilution ensured a countable number of colony-forming units, after plating a suspension of 33 µL.

### 4.2. Irradiation Experiments

**Figure 4:**
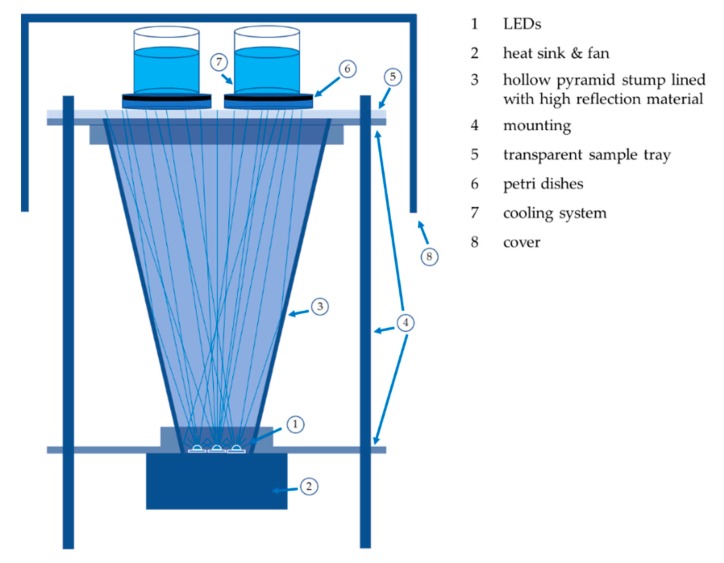
Irradiation set-up: The LEDs were cooled by a heat sink and the radiation was emitted into a pyramid stump with a reflective coating. The resulting homogenized radiation illuminated the legionella, which were incorporated into the cooled agar plates.

The irradiation experiments for the wavelengths 450, 470, and 620 nm were carried out with nearly identical irradiation set-ups, as shown in Figure 4. The LEDs were placed at the pointed end of a hollow pyramid stump lined with high reflection material to irradiate an area of 140 mm × 140 mm with an irradiance of 12.5 mW cm^−2^ (450 and 470 nm set-up) and 25 mW cm^−2^ (620 nm set-up). This allowed the simultaneous irradiation of four agar plates with plated legionella samples. The irradiance was checked and adjusted before and after each irradiation with a power monitor (OPM 150 Qioptiq, Goettingen, Germany). One set-up contained seven high-performance LEDs with a measured emission peak at 445 nm (GD CSSRM2.14-A-RAT-24-1 OSRAM Opto Semiconductors, Regensburg, Germany), which matches the absorption peak of flavins. The 470 nm irradiation set-up included six high power LEDs with a peak at 470 nm (NCSB119 W Nichia, Tokyo, Japan), while the 620 nm irradiation set-up included four high power LEDs (LE A P2W OSRAM Opto Semiconductors, Regensburg, Germany) with an emission peak at 622 nm. The half-width of the LED emissions per assembly was less than 20 nm and the assemblies showed small fluctuations of less than 6% in the irradiation homogeneity.

Different doses were applied for the irradiation experiments, which required different irradiation times at a constant irradiance. A maximum of 300 J cm^−2^ was used for the 450 nm irradiation, while fluences of up to 500 J cm^−2^ were applied to *L. rubrilucens* for 470 and 620 nm irradiation. As shown in Figure 4, the culture media were cooled during irradiation with ice water to keep the temperature below 15 °C. The petri dishes plated with legionella were oriented downwards during irradiation.

### 4.3. Colony Quantification

To determine the antibacterial effect of visible light irradiation, the number of colony-forming units (CFU) was determined. At the end of the irradiation process, the samples were incubated in a humid environment at 37 °C for 5 days. Subsequently, the samples were incubated at room temperature (~20 °C) for another 30 days and compared to the colony numbers after 5 days to investigate the legionella recovery properties.

For each irradiation experiment, adequate dilutions of the three bacterial samples were respectively applied to four agar plates. The same procedure was conducted for non-irradiated controls.

For each applied dose at each wavelength, three independent experiments were carried out—each with four agar plates—and at least three out of these four plates of each individual experiment were evaluated manually. The CFU reduction was determined by the ratio of the arithmetic mean values of irradiated samples and unirradiated controls.

Additionally, the growth of *L. rubrilucens* colonies on unirradiated agar reference plates and irradiated sample plates was documented photographically every 24 h for the highest doses at each irradiation wavelength until colonies became visible on all samples.

### 4.4. Spectroscopic Investigations

For gaining information on the involved photosensitizers, absorption and fluorescence measurements were performed in a legionella lysate similar to the procedure described in [21]. The lysate absorption was measured in a Specord Plus spectrometer (Analytik Jena, Jena, Germany). The lysate fluorescence was recorded by applying two different laser sources: a 405 nm Flexpoint laser (Laser Components, Olching, Germany) or a blue 465 nm LDMC-470-2600 laser (Lasertack, Kassel, Germany). By means of a light probe (Ocean Optics, Largo, FL, USA), the laser light was coupled to the lysate and the fluorescence was detected by a SensLine AvaSpec-2048 XL spectrometer (Avantes, Appelsdorn, The Netherlands).

The lysate spectra were compared to absorption and fluorescence spectra of pure protoporphyrin IX, coproporphyrin III, uroporphyrin III, zinc protoporphyrin, and riboflavin performed with the same instruments.

## Figures and Tables

**Table 1:** Applied irradiation doses, log reduction, and calculated standard deviation of the log reduction for 450, 470, and 620 nm experiments.

Wavelength (nm)	Irradiation Dose (J cm^−2^)	Log Reduction	Standard Deviation of Log Reduction
**450**	50	0.243	0.123
	100	0.565	0.044
	150	1.162	0.107
	200	2.255	0.556
	250	4.317	0.258
	300	5.243	0.648
**470**	50	0.137	0.353
	100	0.249	0.110
	150	0.519	0.210
	200	0.788	0.434
	250	1.267	0.467
	500	5.633	0.425
**620**	100	−0.082	0.050
	200	0.019	0.067
	300	−0.034	0.133
	400	0.000	0.002
	500	−0.027	0.144
